# Synthesis and diverse biological activity profile of triethylammonium isatin-3-hydrazones

**DOI:** 10.5599/admet.1179

**Published:** 2022-01-12

**Authors:** Andrei Bogdanov, Olga Tsivileva, Alexandra Voloshina, Anna Lyubina, Syumbelya Amerhanova, Ekaterina Burtceva, Sergey Bukharov, Alexander Samorodov, Valentin Pavlov

**Affiliations:** 1 Arbuzov Institute of Organic and Physical Chemistry, FRC Kazan Scientific Center of RAS, Kazan, 420088, Russian Federation; 2 Institute of Biochemistry and Physiology of Plants and Microorganisms, Saratov Scientific Centre of the Russian Academy of Sciences, Saratov 410049, Russian Federation; 3 Kazan National Research Technological University, Kazan 420015, Russian Federation; 4 Bashkir State Medical University, Ufa 450000, Russian Federation

**Keywords:** isatin, hemostasis, phytopathogens, antibacterial and antifungal activities

## Abstract

A series of biorelevant triethylammonium isatin hydrazones containing various substituents in the aromatic fragment have been synthesized. Their structure and composition were confirmed by NMR- and IR-spectroscopies, mass-spectrometry and elemental analysis. It was found that some representatives show activity against *Staphylococcus aureus* and *Bacillus cereus* higher or at the level of norfloxacin, including methicillin-resistant *Staphylococcus aureus* strains. The study also showed low hemo- and cytotoxicity (Chang Liver) and high antiaggregatory and anticoagulant activity of these compounds. The high potential of new ammonium isatin-3-acylhydrazones in the search for antimicrobial activity against phytopathogens of bacterial and fungal nature has been shown for the first time.

## Introduction

**Figure 1.**
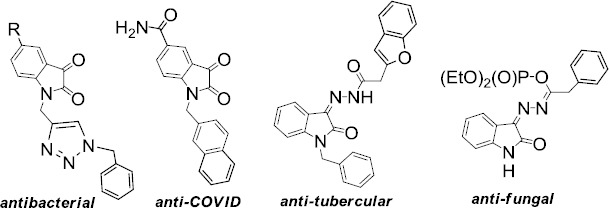
Biologically active isatins and isatin-3-acylhydrazones

As a representative of the class of privileged structures, isatin and its derivatives are widely used in medicinal chemistry [1,2]. The progressing number of works on synthetic procedures [3] and the studies of this heterocycle push researchers in this area to publish generalized data on one or another type of biological activity [4-7]. It is due to the manifestation of isatin and its derivatives of a wide spectrum of activity, such as anti-cancer [8-10], anti-tubercular [11,12], antibacterial [13], anti-COVID [14,15], fungicidal [16,17], etc (Fig. 1).

**Figure 2.**
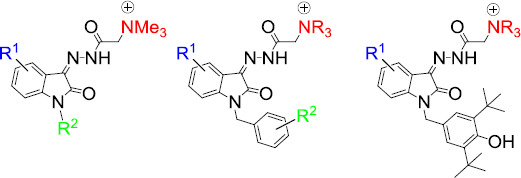
Antimicrobial ammonium isatin-3-acylhydrazones

One of the modern trends in the design and targeted synthesis of bioactive isatin derivatives is the concept of molecular hybridization [18-21]. In this regard, the combination of a pharmacophore fragment and a quaternized nitrogen atom in one molecule seems to be promising in terms of the search for effective and non-toxic biorelevant drugs [22,23]. In recent years, our research group has been working in this direction, namely in the synthesis and study of the antimicrobial activity of water-soluble isatin hydrazones containing a positively charged nitrogen atom (Fig. 2) [24-30]. Those studies showed the dependence of antimicrobial activity on many structural factors. Thus, in the series of trimethylammonium isatin acylhydrazones, sterically hindered analogs showed the best activity against some Gram-positive bacteria [24-26]. We also found that both an increase in the lipophilicity of the benzyl substituent [30] and the presence of an alkyl chain of medium length (C_10_-C_12_) [27] in position 1 leads to an improvement in antimicrobial activity. It is important that all previously obtained compounds have low hemo- and cytotoxic effects.

## Experimental

### Materials and methods

Starting isatins **1a-g** were synthesized accordingly to our previously reported procedure [24]. IR spectra were measured with a Bruker Vector-22 instrument for the samples in KBr pellets. ^1^H and ^13^C NMR spectra were recorded on a Bruker Avance-400 or Bruker Avance-600 Bruker spectrometers at 400, 600 and 100.6, 150 MHz, respectively. Chemical shifts were reported in ppm relative to residual signals of deuterated solvents. CDCl_3_, DMSO-d_6_ or a mixture of CDCl_3_/DMSO-d_6_ were used as the NMR solvents. MALDI mass spectra were recorded on an UltraFlex III TOF/TOF mass spectrometer in linear mode with a recording of positive ions, metal target, and p-nitroaniline matrix. Elemental analysis was performed on a Euro Vector 2000 CHNS-3 instrument; halogen content was determined by pyrolysis in the oxygen stream. Melting points were determined using an SMP10 Stuart instrument and uncorrected.

N,N,N-Triethyl-2-hydrazinyl-2-oxoethanammonium bromide (**1**). White crystalline powder. Yield: 73 %; m.p. 135 °C. IR (KBr, cm^–1^): 3298 (N-H), 3191 (N-H), 3010 (C-H), 2946 (C-H), 1680 (C=O), 1646 (C=O), 1530 (N-H); 1449 (C-N). ^1^H NMR (600 MHz, DMSO-d_6_, δ, ppm): 1.23 t (9H, CH_3_, Et, J 7.2 Hz); 3.43 q (6H, CH_2_, Et, J 7.2 Hz); 3.98 s (2H, CH_2_CO); 4.51 br. s [2H, NH_2_]; 9.89 s (1H, NH). ^13^C NMR (150 MHz, DMSO-d_6_, δ, ppm): 160.90 (C=O); 55.19 (CH_2_); 54.24 (CH_2_); 8.45 (CH_3_). MS (MALDI): 213 [M+K-Br]^+^. Found, %: C, 37.67; H, 7.84; Br, 31.32; N, 16.46. C_8_H_20_BrN_3_O. Calculated, %: C, 37.80; H, 7.93; Br, 31.44; N, 16.53.

### General procedure for the synthesis of compounds 3a-g.

A mixture of substituted isatin **1a-g** (1 mmol) and hydrazide **2** (1 mmol) was magnetically stirred in absolute ethanol (7 mL) for 10 min, followed by the addition of trifluoroacetic acid (20 mol%). Then the reaction mixture was heated at reflux for 3 h. After cooling the solution to room temperature, the solvent was rotary evaporated. The formed precipitate was washed with anhydrous diethyl ether, filtered off and dried in vacuum (12 mmHg).

#### 2-(2-(1-(3,5-Di-tert-butyl-4-hydroxybenzyl)-2-oxoindolin-3-ylidene)hydrazinyl)-N,N,N-triethyl-2-oxoethanammonium bromide (3a).

Yellow powder. Yield: 92 %; m.p. 212 °C. IR (KBr, cm^–1^): 3631 (O-H), 3232 (N-H), 2950 (C-H), 1713 (C=O), 1673 (C=O), 1617 (C=C), 1469 (C=N). ^1^H NMR (600 MHz, CDCl_3_, δ, ppm): 1.36 s (18H, CH_3_, t-Bu); 1.44 t (9H, CH_3_, Et, J 6.9 Hz); 3.88 q (6H, CH_2_, Et, J 7.0 Hz); 4.75 s [2H, NCH_2_]; 5.02 s (2H, CH_2_CO); 5.17 s (1H, OH); 6.84 d (1H, 7-H, Ar, J 7.8 Hz); 8.02-8.03 m (3H, 5-H, Ar, 2H, benzyl); 7.29 dd (1H, 6-H, Ar, J 7.6 Hz, J 7.6 Hz); 8.03 d (1H, 4-H, Ar, J 7.2 Hz); 12.83 s (1H, NH). ^13^C NMR (150 MHz, CDCl_3_, δ, ppm): 165.51 (C=O); 160.90 (C=O); 153.50 (C-OH); 143.49; 136.51; 136.38; 132.07 (CH); 125.25; 124.49 (CH); 123.83 (CH); 123.30 (CH); 118.77; 109.77 (CH); 55.19 (CH_2_); 54.24 (CH_2_); 43.84 (CH_2_); 34.15 (C, t-Bu); 30.06 (CH_3_, t-Bu); 8.45 (CH_3_). MS (MALDI): 521 [M-Br]^+^. Found, %: C, 61.79; H, 7.45; Br, 13.16; N, 9.25. C_31_H_45_BrN_4_O_3_. Calculated, %: C, 61.89; H, 7.54; Br, 13.28; N, 9.31.

#### 2-(2-(1-(3,5-Di-tert-butyl-4-hydroxybenzyl)-7-methyl-2-oxoindolin-3-ylidene)hydrazinyl)-N,N,N-triethyl-2-oxoethanammonium bromide (3b).

Yellow powder. Yield: 97 %; m.p. 154 °C. IR (KBr, cm^–1^): 3634 (O-H), 3204 (N-H), 2959 (C-H), 1714 (C=O),1683 (C=O), 1606 (C=C), 1446 (C=N). ^1^H NMR (400 MHz, CDCl_3_, δ, ppm): 1.36 s (18H, CH_3_, t-Bu); 1.48 t (9H, CH_3_, Et, J 6.0 Hz); 2.35 s (3H, Ar-CH_3_); 3.91 q (6H, CH_2_, Et, J 6.2 Hz); 5.06-5.07 m [4H, NCH_2_, CH_2_CO); 5.14 br. s (1H, OH); 6.97-7.00 m (3H, 6-H, Ar, 2H, benzyl); 7.08-7.11 m (1H, 5-H, Ar); 8.03 d (1H, 4-H, Ar, J 7.0 Hz); 12.91 s (1H, NH). MS (MALDI): 535 [M-Br]^+^. Found, %: C, 62.30; H, 7.56; Br, 12.81; N, 9.01. C_32_H_47_BrN_4_O_3_. Calculated, %: C, 62.43; H, 7.69; Br, 12.98; N, 9.10.

#### 2-(2-(1-(3,5-Di-tert-butyl-4-hydroxybenzyl)-7-ethyl-2-oxoindolin-3-ylidene)hydrazinyl)-N,N,N-triethyl-2-oxoethanammonium bromide (3c).

Yellow powder. Yield: 89 %; m.p. 163 °C. IR (KBr, cm^–1^): 3609 (O-H), 3392 (N-H), 3208 (N-H), 2957 (C-H), 1682 (C=O), 1606 (C=C), 1460 (C=N), 1436 (C=N). ^1^H NMR (600 MHz, DMSO-d_6_, δ, ppm): 0.93 t (3H, CH_3_, Et, J 7.3 Hz); 1.27-1.29 m (27H, CH_3_, t-Bu, CH_3_, Et); 2.63 q (2H, CH_2_, Et, J 7.3 Hz); 3.57-3.62 m (6H, CH_2_, Et); 4.58 s (1H, OH); 4.80 s (2H, CH_2_CO); 5.06 s [2H, NCH_2_]; 6.93 s (2H, 2H, benzyl); 7.13-7.17 m (1H, 5-H, Ar); 7.27 d (1H, 7-H, Ar, J 7.1 Hz); 7.65-7.69 m (1H, 4-H, Ar); 12.64 s (1H, NH). ^13^C NMR (150 MHz, DMSO-d_6_, δ, ppm): 166.21 (C=O); 161.45 (C=O); 152.96 (C-OH); 140.48; 139.70; 134.41 (CH); 127.73; 127.19; 123.63 (CH); 122.01 (2CH); 119.64; 118.99; 54.18 (CH_2_); 53.15 (CH_2_); 44.63 (CH_2_); 34.41 (C, t-Bu); 30.15 (CH_3_, t-Bu); 23.35 (CH_2_); 15.56 (CH_3_); 7.49 (CH_3_). MS (MALDI): 549 [M-Br]^+^. Found, %: C, 62.80; H, 7.73; Br, 12.59; N, 8.78. C_33_H_49_BrN_4_O_3_. Calculated, %: C, 62.95; H, 7.84; Br, 12.69; N, 8.90.

#### 2-(2-(1-(3,5-Di-tert-butyl-4-hydroxybenzyl)-6,7-dimethyl-2-oxoindolin-3-ylidene)hydrazinyl)-N,N,N-triethyl-2-oxoethanammonium bromide (3d).

Yellow powder. Yield: 94 %; m.p. 187 °C. IR (KBr, cm^–1^): 3415 (O-H), 3199 (N-H), 2952 (C-H), 1707 (C=O), 1678 (C=O), 1611 (C=C), 1463 (C=N), 1435 (C=N). ^1^H NMR (600 MHz, CDCl_3_, δ, ppm): 1.34 s (18H, CH_3_, t-Bu); 1.46 t (9H, CH_3_, Et, J 7.3 Hz); 2.18 s (3H, Ar-CH_3_); 2.24 s (3H, Ar-CH_3_); 3.89 q (6H, CH_2_, Et, J 7.3 Hz); 4.97 s [2H, CH_2_CO); 5.05 s [2H, NCH_2_); 5.13 br. s (1H, OH); 6.93-6.95 m (3H, 5-H, Ar, 2H, benzyl); 7.77 d (1H, 4-H, Ar, J 7.1 Hz); 12.83 s (1H, NH). ^13^C NMR (150 MHz, CDCl_3_, δ, ppm): 165.31 (C=O); 162.66 (C=O); 153.10 (C-OH); 143.55; 142.00; 136.57; 136.41; 126.67; 125.90 (CH); 122.61 (CH); 120.54; 120.15; 117.67; 55.26 (CH_2_); 54.22 (CH_2_); 45.53 (CH_2_); 34.23 (C, t-Bu); 30.12 (CH_3_, t-Bu); 21.36 (CH_3_); 13.85 (CH_3_); 8.50 (CH_3_). MS (MALDI): 535 [M-Br]^+^. Found, %: C, 62.35; H, 7.50; Br, 12.83; N, 8.99. C_32_H_47_BrN_4_O_3_. Calculated, %: C, 62.43; H, 7.69; Br, 12.98; N, 9.10.

#### 2-(2-(1-(3,5-Di-tert-butyl-4-hydroxybenzyl)-4-bromo-2-oxoindolin-3-ylidene)hydrazinyl)-N,N,N-triethyl-2-oxoethanammonium bromide (3e).

Yellow powder. Yield: 89 %; m.p. 194 °C. IR (KBr, cm^–1^): 3592 (O-H), 3367 (N-H), 3199 (N-H), 2952 (C-H), 1686 (C=O), 1607 (C=C), 1447 (C=N), 1433 (C=N). ^1^H NMR (400 MHz, CDCl_3_, δ, ppm): 1.37 s (18H, CH_3_, t-Bu); 1.48 t (9H, CH_3_, Et, J 6.9 Hz); 3.90 q (6H, CH_2_, Et, J 7.0 Hz); 4.78 s [2H, NCH_2_]; 4.87 s (2H, CH_2_CO); 5.22 s (1H, OH); 6.88 d (1H, 7-H, Ar, J 7.8 Hz); 7.09 s (2H, 2H, benzyl); 7.18 dd (1H, 6-H, Ar, J 7.8 Hz, J 7.8 Hz); 7.26 d (1H, 5-H, Ar, J 7.8 Hz); 12.93 s (1H, NH). ^13^C NMR (100.6 MHz, CDCl_3_, δ, ppm): 165.37 (C=O); 160.07 (C=O); 153.42 (C-OH); 144.64; 136.33; 134.60; 132.27 (CH); 128.00 (C-H); 124.67; 124.34 (CH); 117.79; 117.09; 108.87 (CH); 65.40 (CH_2_); 55.64 (CH_2_); 43.87 (CH_2_); 33.94 (C, t-Bu); 29.87 (CH_3_, t-Bu); 8.39 (CH_3_). MS (MALDI): 601 [M-Br]^+^. Found, %: C, 54.60; H, 6.40; Br, 23.33; N, 8.11. C_31_H_44_Br_2_N_4_O_3_. Calculated, %: C, 54.71; H, 6.52; Br, 23.48; N, 8.23.

#### 2-(2-(1-(3,5-Di-tert-butyl-4-hydroxybenzyl)-5-nitro-2-oxoindolin-3-ylidene)hydrazinyl)-N,N,N-triethyl-2-oxoethanammonium bromide (3f).

Yellow powder. Yield: 89 %; m.p. 176 °C. IR (KBr, cm^–1^): 3621 (O-H), 3392 (N-H), 3215 (N-H), 2954 (C-H), 1693 (C=O), 1617 (C=C), 1523 (N=O), 1486 (C=N), 1434 (C=N). ^1^H NMR (400 MHz, CDCl_3_, δ, ppm): 1.38 s (18H, CH_3_, t-Bu); 1.49 t (9H, CH_3_, Et, J 6.3 Hz); 3.86-3.90 m (6H, CH_2_, Et); 4.84 s [2H, NCH_2_]; 5.22 s (1H, OH); 5.28 s (2H, CH_2_CO); 6.94 d (1H, 7-H, Ar, J 8.5 Hz); 7.09 s (2H, 2H, benzyl); 8.18 d (1H, 6-H, Ar, J 8.5 Hz); 8.90 br. s (1H, 4-H, Ar); 12.67 s (1H, NH). ^13^C NMR (150 MHz, DMSO-d_6_, δ, ppm): 167.15 (C=O); 161.62 (C=O); 153.52 (C-OH); 148.20; 143.26; 139.51; 127.92 (C-H); 125.94; 124.28 (2CH); 119.59; 116.21; 110.96 (CH); 54.09 (CH_2_); 53.90 (CH_2_); 43.54 (CH_2_); 34.41 (C, t-Bu); 30.20 (CH_3_, t-Bu); 7.49 (CH_3_). MS (MALDI): 566 [M-Br]^+^. Found, %: C, 57.42; H, 6.70; Br, 12.21; N, 10.70. C_31_H_44_BrN_5_O_5_. Calculated, %: C, 57.58; H, 6.86; Br, 12.36; N, 10.83.

#### 2-(2-(1-(3,5-Di-tert-butyl-4-hydroxybenzyl)-5-chloro-7-bromo-2-oxoindolin-3-ylidene)hydrazinyl)-N,N,N-triethyl-2-oxoethanammonium bromide (3g).

Yellow powder. Yield: 89 %; m.p. 177 °C. IR (KBr, cm^–1^): 3629 (O-H), 3400 (N-H), 3209 (N-H), 2956 (C-H), 1689 (C=O), 1606 (C=C), 1447 (C=N). ^1^H NMR (400 MHz, CDCl_3_/DMSO-d_6_, δ, ppm): 1.29 s (18H, CH_3_, t-Bu); 1.33 t (3H, CH_3_, Et, J 7.1 Hz); 3.66-3.70 m (6H, CH_2_, Et); 4.87 s (2H, CH_2_CO); 5.22 s [2H, NCH_2_]; 7.04 s (2H, 2H, benzyl); 7.51 s (1H, OH); 7.68-7.69 m (1H, 6-H, Ar); 7.88 br. s (1H, 4-H, Ar); 12.58 s (1H, NH). ^13^C NMR (100.6 MHz, CDCl_3_/DMSO-d_6_, δ, ppm): 166.14 (C=O); 161.24 (C=O); 153.59 (C-OH); 139.41; 138.11; 136.48 (C-H); 129.29; 127.30; 126.76; 123.98 (C-H); 123.43; 121.22 (C-H); 103.72; 54.90 (CH_2_); 54.13 (CH_2_); 44.49 (CH_2_); 34.60 (C, t-Bu); 30.39 (CH_3_, t-Bu); 8.15 (CH_3_). MS (MALDI): 635 [M-Br]^+^. Found, %: C, 51.90; H, 5.91; Br, 22.23; Cl, 4.72; N, 7.71. C_31_H_43_Br_2_ClN_4_O_3_. Calculated, %: C, 52.08; H, 6.06; Br, 22.35; Cl, 4.96; N, 7.84.

### Antimicrobial activity study

The antimicrobial activity of the test compounds was determined by the serial dilution technique in Muller Hinton Broth 2 and in Sabouradu broth for fungi. The cultures used for testing included Gram-positive bacteria: *Staphylococcus aureus ATCC 6538P FDA 209P*, *Bacillus cereus ATCC 10702 NCTC 8035, Enterococcus faecalis* ATCC *29212*; Gram-negative bacteria: *Escherichia coli ATCC 25922*, *Pseudomonas aeruginosa* ATCC 9027, and fungi: *Trichophyton mentagrophytes* var. gypseum 1773, and *Candida albicans ATCC 10231*; methicillin-resistant strains of *S. aureus* (MRSA) was obtained from hospital patients with chronic tonsillitis in the Republican Clinical Hospital (Kazan, Russia). The bacterial load was 3.0 × 10^5^ cfu/ml. The fungi load was 2.0 × 10^3^ cfu/ml. The results were recorded every 24 h for 5-7 days. Cultures were incubated at 37 °C. The experiment was repeated three times. The dilutions of the compounds were prepared immediately in nutrient media; 5 % DMSO was added for better solubility and the test strains were not inhibited at this concentration. The minimum inhibitory concentration (MIC) was defined as the minimum concentration of a compound that inhibits the growth of the corresponding test microorganism. The growth of bacteria, as well as the absence of the growth due to the bacteriostatic action of compounds, were recorded. To determine minimal bactericidal concentration (MBC), an aliquot of the bacterial culture was transferred onto Mueller-Hinton agar or Sabouradu agar in a 10-cm Petri dish and incubated for 24 h at 37 °C. MBC was the minimal concentration at which bacterial colonies were not detected, indicating that the bacteria were killed with an efficiency of >99.9 % [31].

### Hemolytic activity

Hemolytic activity of test compounds was estimated by comparing the optical density of a solution containing the test compound with that of blood at 100 % hemolysis. The experiments were carried out as described earlier [32].

### Cytotoxicity assay

Cytotoxic effects of the test compounds on human normal cells were estimated by means of the multifunctional Cytell Cell Imaging system (GE Health Care Life Science, Sweden) using the Cell Viability Bio App which precisely counts the number of cells and evaluates their viability from fluorescence intensity [32]. Two fluorescent dyes that selectively penetrate the cell membranes and fluoresce at different wavelengths were used in the experiments. DAPI is able to penetrate intact membranes of living cells and colors nuclei in blue and Propidium iodide dye penetrates only dead cells with damaged membranes, staining them in yellow. DAPI and propidium iodide were purchased from Sigma. IC_50_ was calculated using an online tool: MLA-“Quest Graph™ IC50 Calculator.” AAT Bioquest, Inc, 15 July, 2021, https://www.aatbio.com/tools/ic50-calculator. Chang liver cell line (Human liver cells) from N. F. Gamaleya Research Center of Epidemiology and Microbiology was used in the experiments. The cells were cultured in a standard Eagle’s nutrient medium manufactured at the Chumakov Institute of Poliomyelitis and Virus Encephalitis (PanEco company) and supplemented with 10 % fetal calf serum and 1 % nonessential amino acids. The cells were plated into a 96-well plate (Nunc) at a concentration of 1×10^5^ cells/mL, 150 μL of medium per well, and cultured in a CO_2_ incubator at 37 °C. Twenty-four hours after seeding the cells into wells, the compound under study was added at a preset dilution, 150 μL to each well. The dilutions of the compounds were prepared immediately in nutrient media; 5 % DMSO that does not induce inhibition of cells at this concentration was added for better solubility. The experiments were repeated three times. Intact cells cultured in parallel with experimental cells were used as a control.

### Anticoagulant and anti-aggregation activities study

The in vitro experiments were performed using the blood of healthy male donors aged 18-24 years (total 54 donors). The study was approved by the Ethics Committee of Federal State Budgetary Educational Institution of Higher Education at the Bashkir State Medical University of the Ministry of Health of Russian Federation (No.2 dated 17.10.2012). Informed consent was obtained from all participants before blood sampling. The blood was collected from the cubital vein using the system of vacuum blood collection BD Vacutainer^®^ (Becton, Dickinson and Company, USA). A 3.8 % sodium citrate solution in a 9:1 ratio was used as a venous blood stabiliser. The study of the effect on platelet aggregation was performed using the Born method [33] using the aggregometer «AT-02» (SPC Medtech, Russia). The assessment of antiplatelet activity of the studied compounds and reference preparations was started with the final concentration of 2×10^–3^ mol/L. Adenosine diphosphate (ADP; 20 μg/mL) and collagen (5 mg/mL) manufactured by Tehnologia-Standart Company, Russia, were used as inducers of aggregation. The study on the anticoagulant activity was performed by standard recognised clotting tests using the optical two-channel automatic analyser of blood coagulation Solar CGL 2110 (CJSC SOLAR, Belarus). The following parameters were studied: activated partial thromboplastin time (APTT), prothrombin time (PT) and fibrinogen concentrations according to the Clauss method. The determination of anticoagulant activity of the studied compounds and reference preparation was performed in a concentration of 5×10^–4^ g/mL using the reagents manufactured by Tehnologia-Standart Company (Barnaul, Russia). The results of the study were processed using the statistical package Statistica 10.0 (StatSoft Inc, USA). The Shapiro–Wilk’s test was used to check the normality of actual data distribution. The form of distribution of the data obtained differed from the normal one; therefore, non-parametric methods were used for further analysis. The data were presented as medians and 25 and 75 percentiles. Analysis of variance was conducted using Kruskal–Wallis test. A p value of 0.05 was considered statistically significant.

### Antiphytopathogenic activity study

Plant pathogenic bacterial strains *Micrococcus luteus* B-109, *Pectobacterium atrosepticum* 1043, *Pectobacterium carotovorum* subsp. *carotovorum* MI, *Pseudomonas fluorescens* EL-2.1, and *Xanthomonas campestris* B-610 and fungal strain *Fusarium oxysporum* IBPPM 543 were obtained from the specialized scientific culture collection of IBPPM RAS (WFCC no. 975, WDCM no. 1021) (CM IBPPM). Pathogenic fungus *Phytophthora cactorum* VKM F-985 provided by the All-Russian Collection of Microorganisms (VKM) and deposited at A.E. Favorsky Irkutsk Institute of Chemistry, SB RAS, was also used as test organism. Bacteria *M. luteus*, *P. carotovorum* subsp. *carotovorum*, *P. atrosepticum*, and *Ps. fluorescens* were grown in meat-peptone medium (BP), and *X. campestris* was grown in the medium with glucose, yeast extract, and calcium carbonate (GYCa). Solid media contained Bacto agar (18 g/L); pH was adjusted to 7.2-7.4. All bacterial cultures were grown at 28°C. The mycelial cultures of *F. oxysporum* and *P. cactorum* were grown on a glucose-peptone-yeast (GPY) nutrient medium at 27 °C. For inoculum preparation, both fungal strains were initially grown on the agar GPY in Petri dishes and then transferred into the seed medium by punching out 5 mm of the agar plate culture with a self-designed cutter.

Antibacterial and antifungal activities of the compounds were explored using the agar diffusion method and the technique of a phytopathogen radial growth inhibition. Method of diffusion in agar (measuring the diameter of growth inhibition zones) was used for determining the bactericidal activity. The 6-mm wells were made in ager medium (GYCa for *Xanthomonas campestris* or BP for other bacteria). Bacterial suspensions were distributed over the agar surface, and the tested compound's solution (150 μl) was added to each well. The width of growth inhibition zones around the wells was determined after incubation for 36-40 h.

For the fungicidal activity analysis, radial growth (colony diameters) of the fungi on a solid medium in the absence and in the presence of the compounds **3a,c-g** solutions at various concentrations were compared. The method consisted of the following: sterile, melted and then cooled to about 60°C GPY agar medium (20.0 ml) was mixed with the precisely measured volumes of the solutions under question, and poured into a sterile Petri dish (90 mm i.d.). After solidification of GPY agar, the media were inoculated by the fungus using 10-day-old cultures of *F. oxysporum* or *P. cactorum*. The inoculation was done by transferring a 5-mm (i.d.) GPY-agar block covered with mycelium to the center of the Petri dish followed by incubation in a thermostat at 27°C. The fungicidal effect was scored by the size of mycelium colony on a Petri dish compared to the control without fungicidal admixtures to GPY agar. Each treatment was performed in at least four replicates in two independent experiments. The observation period ended when the control Petri dish was filled with mycelium (usually after 12 days). The inhibition of the phytopathogen colony growth by the compounds **3a,c-g** or fludioxonil solutions was calculated as a percentage by which the mycelium radial propagation was decreased compared to the unaffected control, the latter was taken as 100 % of growth (or zero percent of inhibition). The EC_50_ value was calculated as the compound concentration at which the radial growth of the fungus colony was decreased by 50 % relative to the non-fungicidal control, according to the formula EC_50_ = 50C/I based on the approach developed e.g., in [34,35].

The solutions of tested compounds were prepared with a concentration of 2 mmol/l (stock solution). For comparison, test compounds were also commonly applied disinfecting agents and antibiotics.

## Results and Discussion

### Chemistry

The synthesis of target compounds is based on our previously developed approach [24-30] and is carried out in two stages (Fig. 1). At the first stage, hydrazide **1** (an analog of the Girard’s reagent T) was obtained for the first time, containing three ethyl groups at the quaternized nitrogen atom. Further, this hydrazide was involved in the condensation with isatin derivatives bearing a phenolic fragment in position 1 and substituents of different nature in the benzo fragment of the heterocycle. The reaction proceeds in ethanol at reflux temperature for 3 hours in the presence of trifluoroacetic acid as a catalyst. After easy workup, the desired reaction products were isolated in pure form with high yields (89-97 %). The structure of all obtained hydrazones **3a-g** was proved using NMR and IR spectroscopy. Thus, the IR spectra of the new compounds contain narrow, intense absorption bands in the region of 3600-3630 cm^-1^ and broad bands of medium intensity at 3210-3380 cm^-1^, corresponding to the stretching vibrations of O-H and N-H bonds, respectively. The presence of these functional groups is also confirmed by the data of ^1^H NMR spectra, in which the signal of the proton of the hydroxyl group appears in the region of ~ 5.2 ppm, and the signal of the hydrazone proton in the lowest fields at ~ 12.8 ppm.

### Antimicrobial activity evaluation

Synthesized compounds were further evaluated for antimicrobial activity against test microorganisms: *Staphylococcus aureus* ATCC 209p (*Sa*), *Bacillus cereus* ATCC 8035 (*Bc*), *Escherichia coli* CDC F-50 (*Ec*), *Enterococcus faecalis* (*Ef*), *Pseudomonas aeruginosa* ATCC 9027, *Aspergillus niger* BKMF-1119, *Trichophyton mentagrophytes* var. *gypseum* 1773, and *Candida albicans* 855-653 including methicillin-resistant strains of *S. aureus* (*MRSA 1* and *MRSA 2*). The minimum inhibitory (MIC) and minimum bactericidal (MBC) concentrations of ammonium salts were determined against these pathogens (Table 1). Due to the lack of activity of compounds **3a-g** against Gram-negative bacterial strains and some fungi, the corresponding data are not shown.

The initial study of antimicrobial activity showed that tri*ethyl*ammonium derivatives **3a-g**, like their tri*methyl*ammonium analogs [24-27], selectively act on Gram-positive pathogenic bacterial strains with high activity. Compared with norfloxacin against *Sa*, compounds **3a-e** (MIC 8.9-3.1 μM) turned out to be the best in bacteriostatic effect, and hydrazones **3b,c** (MIC ~25 μM) were the best against *Bc*. According to the bactericidal action derivative **3c** was determined as the leader compound, containing an ethyl radical in position 7 with an MBC of 6.2 μM, better than that of the reference drug. It should be noted that only compound **3d** showed antimicrobial activity against *Enterococcus faecalis* that often causes a wide range of nosocomial human infections. It is especially important to note that three representatives of this series - compounds **3b-d** - showed high activity against both types of *MRSA* used. In this case, the most effective was the most lipophilic hydrazone **3d**, containing two methyl groups in the aromatic fragment of the heterocycle. This compound also showed moderate activity against the yeast-like fungus *Candida albicans*. Thus, the data obtained showed that acylhydrazones bearing electron-donor alkyl groups in the benzo fragment possess the best activity against both museum and methicillin-resistant bacterial strains.

**Figure 3.**
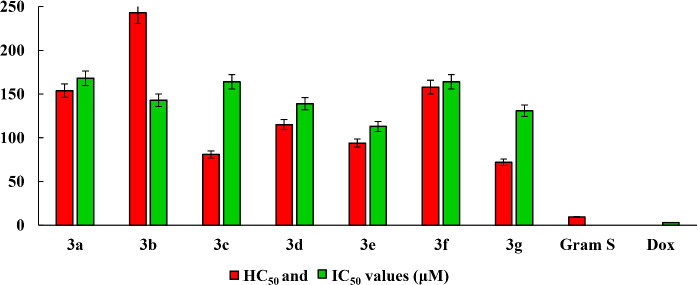
Hemolytic and cytotoxic activity of **3a-g**

An important step in the determination of the biological activity of new chemical compounds is the assessment of their cytotoxic action in relation to mammalian cells. The ability of the investigated compound to cause the destruction of human erythrocytes illustrates its toxic effect on the internal environment of the body. The hemolysis assay is a simple screening test, the results of which can help in the study of cytotoxicity in more complex models. Such experimental models can be the cell lines obtained from various organs and tissues of a person and allowing to adequately assessing the effect of new potential drugs on cell metabolism. In this regard, the studied compounds were tested for cytotoxicity against blood erythrocytes and the human hepatocytes (Chang liver cell lines (Fig. 3). Hemolytic and cytotoxic activity data are represented by HC_50_ and IC_50_ values. It can be seen that all compounds **3a-3g** in the range of tested concentrations did not have high hemolytic and cytotoxic activity. HC_50_ concentrations were 72.2-243.8 μM; IC_50_ - 113-168 μM. From the point of view of the effect on red blood cells, the least toxic compound is compound **3b** containing methyl group at position 7 of the aromatic fragment. Reference drugs gramicidin S and doxorubicin turned out to be much more toxic to red blood cells and liver cells.

**Figure 4.**
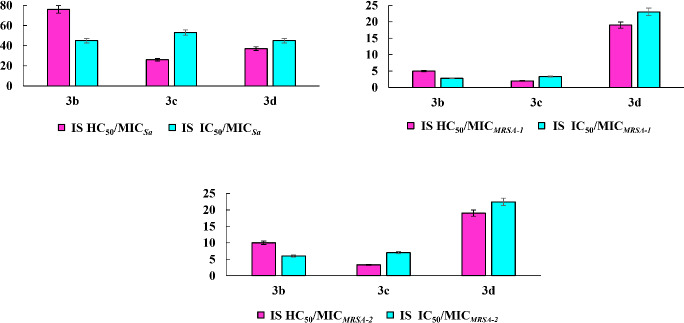
Selectivity of **3b, 3c** and **3d** for bacteria (*S. aureus 209, MRSA-1and MRSA-2)* compared to red blood cells and Chang liver cells.

The selectivity of compounds for microbial cells is an important criterion for assessing the cytotoxic effect. This indicator is characterized by the value of the selectivity index (SI), which for the leading compounds **3b, 3c**, and **3d** was calculated as the ratio between the HC_50_ value for erythrocytes (IC_50_ for eukaryotic cells) and the MIC value for bacterial cells (Fig. 4). It can be seen that with respect to the *S. aureus* 209 P test strain, all tested compounds exhibit a sufficiently high selectivity. Compound **3d** bearing two methyl groups demonstrated the most significant selectivity against resistant strains *MRSA-1* and *MRSA-2* compared to erythrocytes and hepatocytes. Its selectivity index was 3–8 times higher than that of compounds **3b** and **3c**.

### Anticoagulant and anti-aggregation activities evaluation

The development of new drug candidates is a multi-stage process that requires consideration of the influence of many factors, including the level of toxicity and the presence of side effects. There are many examples of drug recalls from the market in world practice, which gradually reveals serious side effects associated, for example, with the risk of developing cardiovascular diseases [36,37]. In this regard, it seemed appropriate and important to assess the effect of the newly synthesized isatin-3-acylhydrazones on the hemostatic system. In this work, we carried out a primary study of the anti-aggregation and anticoagulant activity of compounds **3a-g** (Table 2).

The compounds demonstrated the varying extent of the effect on the plasmatic component of the haemostasis system that manifested only by a change in the parameter APTT of the intrinsic coagulation pathway. Compounds **3c,f** demonstrated anticoagulant activity ≥10 % (p<0.05). Regarding the impact on platelet aggregation, the compounds demonstrated similar activity on both aggregation inductors. Compounds **3a-c,g** demonstrated antiaggregatory activity *in vitro* at the level of acetylsalicylic acid. The most promising active compound is 7-ethyl substituted analog **3c**. Among the obtained series of new compounds, this hydrazone is the most active.

### Antiphytopathogenic activity evaluation

To expand the range of possible practical applications of isatin derivatives, synthesized compounds were examined for their antibacterial and antifungal effects against test organisms. Bacterial pathogens isolated from the plants microenvironment and harvested vegetables frequently include *Micrococcus luteus*, *Pseudomonas fluorescens*, *Pectobacterium carotovorum* (*Erwinia carotovora*), *Xanthomonas campestris*, and *Pectobacterium atrosepticum* (*Erwinia carotovora* subsp. *atrosepticum*) [38-40]. Fungal pathogens cause 70–80 % of all plant diseases [41], possessing a potentiality of causing large-scale disease outbreaks in a very limited period of time. Among the diverse mycelial pathogenic fungi, those from the genera *Fusarium* and *Phytophthora* are among the most destructive plant pathogens known, have broad host ranges, and are capable of causing crop losses and eventual collapse of whole infected plants [42]. *F. oxysporum* is referred to as the aggressive pathogen among *Fusarium* species, and, in particular, different branches of the food industry are extremely conscious of *Fusarium* infections of cereals [43]. *Phytophthora cactorum* is a causative agent of phytophthora blight of ginseng (Panax ginseng), a plant that is very useful for conventional medicine to treat various diseases, including cancer [44].

Along with the solutions of **3a,c-g**, test compounds were sodium hypochlorite (1000 μg/ml), chlorohexidin (500 μg/ml), and norfloxacin (500 μg/ml), a synthetic fluoroquinolone with broad-spectrum antibacterial activity against most bacteria. A commercial widely used fungicide fludioxonil capable of inhibiting the pathogenic fungi mycelium propagation served for the purpose of comparison in the course of the fungicidal effect assays. Under laboratory conditions, the assessment of the phytopathogenic fungi resistance to this fungicide is referred to be conducted at its concentrations from 0.1 to 10 μg/ml [45].

The results of determining the bactericidal activity against *M. luteus* B-109, *P. atrosepticum* 1043, *P. carotovorum* subsp. *carotovorum* MI, *Ps. fluorescens* EL-2.1, *X. campestris* B-610 showed non-zero activity against bacterial test systems of all the compounds tested (Table 3).

Compounds **3a, c-g** showed high antibacterial activity in this experiment. Only in a few cases was the inhibition zone width of 5 and 7.5 mm (**3f** versus *P. carotovorum* and *X. campestris*, respectively), 7 mm (**3g** versus *M. luteus*) found. Other drugs with an even more pronounced bactericidal effect formed the growth of phytopathogens inhibition zone from 8 to 15 mm (Table 4).

Taking into account the data in Table 4, it is quite conditionally possible to arrange the studied 2 mM solutions of compounds **3a,c-g** in decreasing order of activity as follows: **3e**> **3c**> **3a**> **3d**> **3g**> **3f**. Thus, with respect to the phytopathogens used, the best activity was shown by acylhydrazone **3e** containing a bromine atom at position 4 of the heterocycle.

Screening for the manifestation of antifungal properties of compounds **3a,c-g**, introduced into the agar medium to cultivate the fungus *F. oxysporum*, was carried out in the concentration range of 6-43 μg/mL (Table 5).

The antifungal properties of compounds **3a,c-g**, introduced into the agar medium to cultivate the fungus *P. cactorum*, were studied in the concentration range 1-15 μg/mL. The amount of inhibition of fungal growth was expressed as a percentage, taking into account that the quantitative characteristic of the absence of inhibition is expressed as 0 % (table 6).

All compounds **3a,c-g** showed an antagonistic effect against *P. cactorum*, superior to fludioxonil from 2.5 (**3d**) to 13.2 (**3c**) times by seven days of the fungus growth. At the same culture age of *F. oxysporum*, almost all compounds of this series outperformed fludioxonil in antifungal effect by up to 7.9 times (hydrazone **3c**). It is important to note that fludioxonil showed the greatest antifungal effect at the age of both cultures three days, and then the fungicidal effect of the commercial drug was significantly reduced (Tables 5, 6). At the same time, the fungicidal properties of compounds **3e**, **3a**, **3c** increased during the development of the mycelium. The inhibition value I by these drugs after 5-9 days of *P. cactorum* growth increased by a maximum of 27 % (**3e**), in *F. oxysporum* - by 28 % (**3c**). Moreover, in the case of using the *Fusarium* test system, an increase in antifungal ability in the dynamics of the experiment was revealed in the entire group **3a,c-g** (Table 6). In decreasing order of activity against *F. oxysporum*, the following series is obtained: **3c**> **3e**> **3f**> **3a**> **3d**> **3g**. For *P. cactorum*, the series is as follows: **3c** > **3e** > **3g** > **3f** > **3a** > **3d**. Thus, newly synthesized isatin derivatives of **3a,c-g**, possessing significant antibacterial activity, were able to significantly inhibit the growth of pathogenic fungi *F. oxysporum* and *P. cactorum* on a dense medium. First of all, these are 4-bromo- (**3e**) and 7-ethyl derivatives (**3c**) - "leaders" in all sequences of the location of compounds in terms of antiphytopathogenic activity: against pathogenic bacteria, against fungi *F. oxysporum* and *P. cactorum*.

## Conclusions

A number of new isatin-3-acylhydrazones containing a triethylammonium fragment have been synthesized, and their multiple biological profiles were revealed. The study of antimicrobial properties showed that the compounds obtained have selective activity against *Staphylococcus aureus* and *Bacillus cereus*. Derivatives containing donor alkyl substituents showed bacteriostatic activity two times better than norfloxacin, and the 7-ethyl analog was slightly better than the reference drug in terms of its bactericidal effect. The results obtained showed low toxicity of new compounds towards healthy human cells (red blood cells, hepatocytes) and the absence of a negative effect on some factors of hemostasis. The study of antiphytopathogenic activity showed that 4-bromine (**3e**) and 7-ethyl (**3c**) analogs have high activity against a number of dangerous bacterial (*Micrococcus luteus* and *Pectobacterium atrosepticum*) and fungal (*F. oxysporum* and *P. cactorum*) pathogens that exceeds those for reference drugs norfloxacin, chlorohexidine and fludioxonil respectively. Thus, the data obtained indicate a high potential for the development of effective biocompatible drugs for both pharmaceutical and agricultural purposes.

## Figures and Tables

**Scheme 1.**
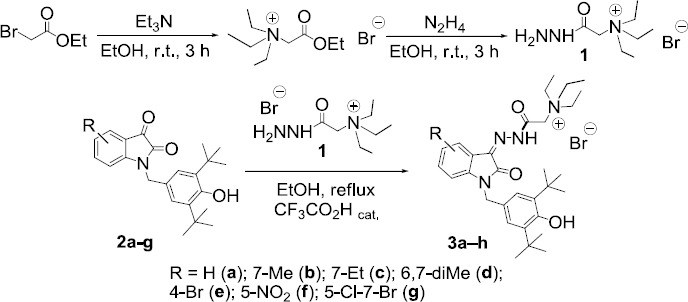
Synthetic route to the ammonium hydrazones **3a-g**

**Table 1.** Antimicrobial activity of compounds 3a-g^*^.

Compound	MIC/MBC, μM					
	*Sa*	*Bc*	*Ef*	*MRSA-1*	*MRSA-2*	*Ca*
**3a**	6.5±0.5/ 13.0±1.2	52±4.7/-	-	nd*	nd	-**
**3b**	3.2±0.2/ 25.4±2.1	25.4±1.9/ 203±16	-	50.8±4.1/ 203.2±16.4	25.4±2.3/ 50.8±4.1	-
**3c**	3.1±0.2 /6.2±0.5	24.8±1.9/ 99.3±8.2	-	49.7±3.9/ 99.3±8.3	24.8±2.2/ 99.3±8.3	-
**3d**	3.1±0.2/ 12.4±1.1	49.6±3.9/ 198.5±15.7	49.6±3.9/ 49.6±3.9	6.2±0.5/ 12.4±1.2	6.2±0.5/ 49.6±3.8	198.5±15.5/-
**3e**	8.9±0.7/ 17.7±1.5	71±6.2/ 283.7±22.7	-	nd	nd	-
**3f**	48.3±3.9/ 96.7±7.6	397±31.7/-	-	nd	nd	-
**3g**	11±0.9/ 21.9±1.8	43.7±3.5/ 87.4±7.2	-	nd	nd	-
**Norfloxacin**	7.5±0.5/ 7.5±0.6	24.4±2.1/ 24.4±2.1	7.5±0.5/ 7.5±0.6	391.4±30/na§	30.0±2.6/na	

* were not determined (compounds possess low activity)

§ no activity

** means >500

**Table 2.** Anticoagulant and anti-aggregation activity of compounds **3a-g**

Compound	Platelets aggregation, % of control		*APTT*^$$^, % of control
	*ADP* ^$^	*Collagen*	
**3a**	-10.5 (8.7-13.9)*	-9.4 (8.5-12.3)*	+5.2 (4.1-7.6)*, †
**3b**	-9.7 (7.4-12.3)*	-10.3 (9.6-12.5)*	+7.3 (6.5-9.6)*, †
**3c**	-13.7 (11.5-15.1)**	-12.5 (9.8-14.3)*	+11.2 (7.9-13.1)*, †
**3d**	-9.0 (7.5-10.6)*, #	-8.5 (7.2-12.3)*, #	+4.8 (3.7-6.9)‡
**3e**	-0.5 (0.2-0.9)##	-2.4 (1.7-3.5)##	+3.2 (2.8-5.4)‡
**3f**	-4.6 (3.7-5.3)*, #	-4.3 (3.4-5.6)*, #	+10.3 (8.5-12.7)*,†
**3g**	-10.2 (8.6-12.2)*	-8.9 (7.5-10.3)*, #	+8.6 (6.1-10.9)*,†
**Acetylsalicylic acid**	-13.7 (10.8-16.4)*	-14.7 (12.1-16.3)**	-
**Heparin sodium**	-	-	+20.3 (19.7-21.4)**

*p ≤ 0.05

**p ≤ 0.001 - compared to control

#p ≤ 0.05

##p ≤ 0.001 - compared to acetylsalicylic acid

†p ≤ 0.05

‡p ≤ 0.001 - compared to Heparin sodium

^$^ ADP – adenosine diphosphate

^$$^ APTT - activated partial thromboplastin time.

**Table 3.** Bactericidal activity of hydrazones (2 mM/L) studied*.

Compound**	Bacterial phytopathogen				
	*M. luteus*	*P. atrosepticum*	*P. carotovorum subsp. carotovorum*	*Ps. fluorescens*	*X. campestris*
**3a**	9	8	10	9	10
**3c**	10.5	9	9	8	11
**3d**	8	8	8	9	8
**3e**	13	15	10	9	11
**3f**	10	10	5	10	7.5
**3g**	7	9	9	8.5	8
**Norfloxacin, 500 μg/mL**	7	7	8	8	7
**sodium hypochlorite, 1000 μg/mL**	2.5	4	3	4	4
**Chlorohexidin, 500 μg/mL**	5	4	5	4	5

* shows the values of the width of the inhibition zone (mm), averaged over the results of 3 experiments

** compound **3b** was not tested due to the formation of precipitate at the solution preparation

**Table 4.** Comparative bactericidal activity of compounds **3a, c-g** (2 mM/L).

Bacterial test system	Inhibition zone width (mm), not less					
	15	13	11	10	9	8
*Micrococcus luteus* B-109	**–**	**3e**	**–**	**3c>3f**	**3a**	**3d**
*Pectobacterium atrosepticum* 1043	**3e**	**–**	**-**	**3f**	**3c,48**	**3a, 3d**
*Pectobacterium carotovorum* subsp. *carotovorum* MI	**–**	**–**	**-**	**3e, 3a**	**3c, 3g**	**3d**
*Pseudomonas fluorescens* EL-2.1	**–**	**–**	**–**	**3f**	**3e, 3a, 3d**	**3g >3c**
*Xanthomonas campestris* B-610	**–**	**–**	**3e, 3c**	**3a**	**-**	**3d, 3g**

**Table 5.** Fungicidal activity of compounds **3a,c-g** against *Fusarium oxysporum* IBPPM 543.

Compound	C, μg/mL	Inhibition value, I (%)*, at the age of the fungus (days)				EC_50_, μg/mL at day 7
		3	5	7	9	
**3a**	6.02	11	4	9	0	33.44
	12.04	14	12	18	3	
	24.08	24	16	27	4	
	36.12	28	24	30	12	
**3c**	6.30	2	11	30	17	10.50
	12.60	24	26	33	23	
	25.20	31	33	47	27	
	37.80	44	42	59	38	
**3d**	6.30	0	5	5	0	48.46
	12.60	8	12	13	2	
	25.20	17	15	20	8	
	37.80	22	18	24	9	
**3e**	6.81	11	13	19	6	17.92
	13.62	14	18	22	9	
	27.24	19	19	25	20	
	40.86	40	37	43	29	
**3f**	6.47	13	11	10	9	30.81
	12.94	26	23	21	10	
	25.88	28	27	32	12	
	38.82	29	28	34	18	
**3g**	7.15	0	4	5	4	55.00
	14.30	9	4	11	5	
	28.60	11	7	26	10	
	42.90	31	27	32	13	
**Fludioxonil**	10	38	23	6	4	83.33

* the average values are given; the standard deviation did not exceed 0.03 from the given value.

**Table 6.** Fungicidal activity of compounds **3a,c-g** against *Phytophthora cactorum* VKM F-985.

Compound	*C*, μg/mL	Inhibition value, *I* (%)*, at the age of the fungus (days)					EC_50_, μg/mL at day 7
		3	5	7	9	12	
**3a**	3.01	4	9	15	1	0	10.03
	6.02	13	21	22	4	5	
	9.03	17	22	25	4	5	
	12.04	40	47	49	41	26	
**3c**	1.26	17	28	30	12	3	2.10
	3.15	29	31	38	20	16	
	6.30	33	44	45	28	20	
	9.45	39	45	48	41	36	
	12.60	50	56	61	58	48	
**3d**	1.26	18	17	5	0	0	11.25
	3.15	23	18	14	6	5	
	6.30	29	19	15	8	7	
	9.45	31	20	17	9	8	
	12.60	50	50	51	48	35	
**3e**	1.36	8	14	22	10	3	3.10
	3.41	13	21	34	12	5	
	6.81	17	33	38	16	7	
	10.22	39	40	53	28	18	
	13.62	45	62	72	70	66	
**3f**	1.29	17	11	2	0	0	8.99
	3.24	33	19	18	1	0	
	6.47	35	23	20	8	1	
	9.71	41	36	32	20	16	
	12.94	59	55	53	48	30	
**3g**	1.43	30	29	15	1	0	4.77
	3.58	38	29	22	5	0	
	7.15	40	33	26	14	11	
	10.73	48	43	41	31	26	
	14.30	51	48	46	41	28	
**Fludioxonil**	10	46	38	18	12	9	27.78

* the average values are given; the standard deviation did not exceed 0.03 from the given value.
